# Increased ZAP70 Is Involved in Dry Skin Pruritus in Aged Mice

**DOI:** 10.1155/2016/6029538

**Published:** 2016-04-18

**Authors:** Nan Zhao, Min Gu, Wenxiu Yang, Man Zhang, Qi Tian, Liyan Ru, Yang Lü, Weihua Yu

**Affiliations:** ^1^Institute of Neuroscience, Department of Human Anatomy, Chongqing Medical University, Chongqing 400016, China; ^2^Department of Geriatrics, The First Affiliated Hospital of Chongqing Medical University, Chongqing 400016, China

## Abstract

Dry skin pruritus is common in the elderly. Recent reports show that T-cell signal path is involved in dry skin pruritus. Zeta-chain-associated protein kinase 70 (ZAP70), as a T-cell receptor, may induce interleukin 2 (IL-2) secretion and promote nerve growth factor (NGF) secretion in skin. This study aimed to detect the alteration of ZAP70 in a mice model with dry skin pruritus. The C57BL mice with 5 months and 22 months were used as experimental animal. Following a 5-day period of treatment of back with a mixture of acetone-diethyl-ether-water (AEW), mice exhibited a significant increase in spontaneous scratching behavior directed to the treated back compared to control animals in which back was similarly treated with water only (W). After AEW process, spontaneous scratching in 22-month AEW mice was increased compared to 5-month AEW mice. Western blot and real-time quantitative PCR data analysis showed that ZAP70 expression was significantly increased in 22-month AEW mice compared with 5-month AEW mice. ELISA data showed that secretions of IL-2 and NGF in 22-month AEW mice were higher than 5-month AEW mice. Our results indicate that increased ZAP70 is involved in dry skin in elderly pruritus. Increased secretion of IL-2 and NGF may induce dry skin itch.

## 1. Introduction

Itch is one of the most important symptoms in inflammatory skin diseases and allergic disorders. It is defined as “unpleasant sensation, eliciting the urge to scratch.” Dry skin is one of the most pruritic skin diseases. It is a primary complaint associated with xerosis and other dermatoses that compromise skin barrier integrity, such as atopic dermatitis and psoriasis [1, 2]. Elderly people are believed to be particularly prone to pruritus with dry skin; these are problems of high relevance in the aged population, especially in the development of irritant contact dermatitis [3]. As the prevalence of atopy continues to rise, it becomes increasingly important that clinicians understand the mechanisms responsible for itch and, in particular, dry skin itch [4]. However, the mechanism of dry skin pruritus is poorly understood. All of importance T cells directly communicate with nerves to regulate neurogenic inflammation of pain and are involved in pruritus as well [5–7]. Thus, immune response may explain the mechanism of dry skin pruritus.

Zeta-chain-associated protein kinase 70 (ZAP70) is a cytosolic protein, and it is recruited at the plasma membrane of T cells following TCR stimulation. It regulates thymocyte development as well as motility, adhesion, and cytokine secretion of mature T cells including interleukin 2 (IL-2) [8]. Also, epidermal hyperinnervation is considered as one cause of itch sensitization and is thought to be regulated by keratinocyte-derived axonal guidance molecules, including nerve growth factor (NGF). These cytokines were explicit and involved in dry skin pruritus [9, 10].

This paper presumes ZAP70 may be involved in dry skin pruritus via promoting cytokines secretion, especially in the elderly. In our study, a mouse model of dry skin itch was used to investigate presumptive mechanism. Behavioral observation shows that spontaneous scratching in elderly AEW mice was much serious than young AEW mice. Data analyses revealed that expression of ZAP70 was significantly increased in elderly AEW group compared to young AEW group. This study shows the alteration of ZAP70 in elderly mice with dry skin pruritus. Our result first shows that increased ZAP70 expression might regulate dry skin pruritus in the elderly mice.

## 2. Materials and Methods

### 2.1. Experimental Animals

All experimental procedures were approved by the Ethics Committees of Chongqing Medical University. All of C57BL mice with elderly group (22 months, *n* = 10, unlimited gender) and young group (5 months, *n* = 10, unlimited gender) from the Experimental Animal Center of Chongqing Medical University were used in the present study.

Acetone-diethyl-ether-water (AEW) model, as an inexpensive and stable model that imitated dry skin condition accurately, has become an internationally recognized animal model of chronic itch. It is proved that AEW model has evidence for the dryness with transepidermal water loss (TEWL) and measuring hydration test [2, 11–15]. AEW model of dry skin pruritus was performed as follows. The hair of mice was shaved over the rostral part of the back at least 3 days before the start of the experiment. Then, we treated the back skin by cotton (3 × 3 cm^2^) soaked with a 1 : 1 mixture of acetone and diethyl-ether (AE) which was laid upon the shaved area for 15 s. After AE treatment, cotton soaked with distilled water was laid upon the same area for 30 s immediately. The treatment was performed twice daily (9:00 and 17:00) under ether anesthesia; such treatment was repeated once daily for 5 days. Cotton soaked with physiological saline solution was laid on the shaved area for 60 s as control group.

### 2.2. Behavioral Observation

Scratching of mice was observed by videotape. Briefly, six mice were put individually into an acrylic box (26 × 18 × 30 cm^3^) composed of six cells. They were acclimated to the experimental environment for at least 30 min, and then behaviors were videotaped with experimenters kept out of the observation room in the morning. Playback served for counting the scratching of the treated area by the hind paws: the mouse generally as one bout of scratching. Thus, we observed scratching of the mouse at least 1 h after the treatment for barrier disruption on the previous day.

### 2.3. Immunohistochemistry

The control and model mice were beheaded after being anesthetized with pentobarbital (1 mg/g, i.p.). After paraffin embedding, the skin tissues were then sectioned at 5 mm thickness for immunohistochemistry. Skin was fixed in 4% paraformaldehyde for 1 h blocked by 1% BSA/PBS for 2 h and then immunostained with a purified polyclonal rabbit antibody ZAP70 (1 : 100, Sigma) and then sections were washed and incubated with the secondary antibody directed against rabbit IgG (1 : 200; Dianova). Following tissue sections were incubated with Streptavidin-HRP. Several visual fields images in a tissue section were collected randomly by the OLYMPUS PM20 automatic microscope (Olympus, Tokyo, Japan).

### 2.4. Western Blotting

Protein concentrations were estimated by the background-corrected absorbance (BCA) method (Beyotime Biotechnology). Tissue samples (5 *μ*L/lane) were run in 10% of Tris-glycine gels depending on the molecular weight of the protein of interest and transferred onto PVDF membranes (Millipore). The PVDF membranes were blocked in 5% bovine serum albumin (Genview) for 2 h. The PVDF membranes were incubated with primary antibodies: rabbit anti-ZP70 antibody (1 : 750, AB319; Sigma-Aldrich, Saint Louis, MO) and mouse anti-*β*-actin as control antibody (1 : 1000, Beyotime, China) followed by incubation with anti-mouse (Beyotime Biotechnology) and anti-rabbit (Beyotime Biotechnology) immunoglobulin conjugated to horseradish peroxidase. Finally, membranes were incubated with enhanced chemiluminescence (ECL) and were analyzed by gel/luminescence image analysis system.

### 2.5. Real-Time Quantitative PCR Analysis of ZAP-70

Total RNA was purified from cell tissues and used for reverse transcription. PCR was performed on ZAP-70 primers (forward primer: 5′-ACGCGCCAGAGTGCATCAA-3′, reverse primer: 5′-CTCGGGGCCCTTCATTTTCTT-3′) or GAPDH internal control primers (forward primer: 5′-GGCCGGTGCTGAGTATGTCGTG-3′, reverse primer: 5′-GGGGGGCTAAGCAGTTGGTGG-3′). The real-time qPCR was performed on a Strata gene Mx3000P instrument.

### 2.6. ELISA

IL-2 and NGF values were assayed with an enzyme-linked immunosorbent assay (ELISA) (mice IL-2 and NGF high sensitivity ELISA with Signal Amplification, eBioscience) in the skin of AEW model mice and control subjects, according to the manufacturer's instructions.

### 2.7. Statistical Analysis

Statistical comparisons were performed with two-way repeated measured ANOVA or Student's *t*-test. All data are expressed as the mean ± SEM. *P* < 0.05 was considered significant.

## 3. Results

### 3.1. Elderly AEW Mice Showed a Serious Pruritus Compared to Young AEW Mice

**Figure 1:**
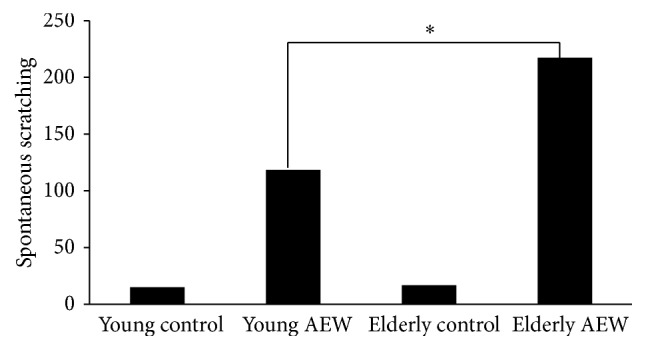
The numbers of spontaneous scratching after 1 hour of AEW treatment. The numbers of scratching were 218 bouts in elderly AEW group and 118 bouts in young AEW group, respectively.  ^*∗*^Represented statistic difference of numbers of scratching between elderly AEW group and young AEW group (*P* < 0.05).

Behavior data analysis in elderly AEW group was significantly increased compared to young AEW group which demonstrates that dry skin pruritus in elderly mice was more serious than in young mice. The average number in 1 hour of elderly AEW group was 218 bouts and the young AEW group was 118 bouts (Figure 1).

### 3.2. The Expression of ZAP70 Was Significantly Increased in Elderly AEW Mice with Dry Skin Pruritus

**Figure 2:**
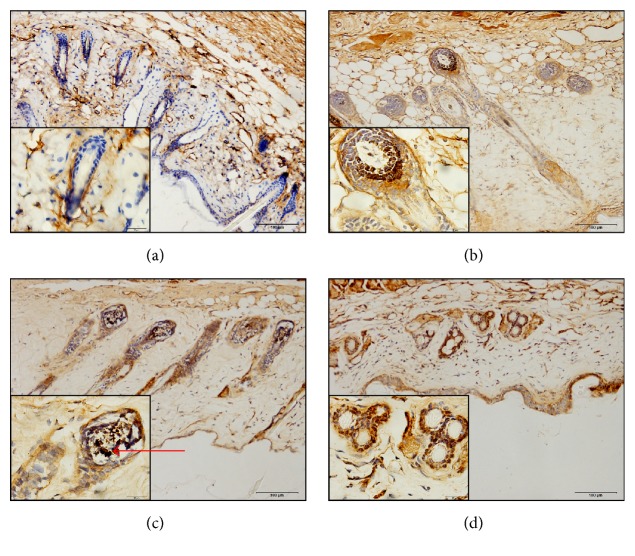
The expression of ZAP70 in the skin. (a) Young control group, (b) young AEW group, (c) elderly control group, and (d) elderly AEW group. The arrow meant enlarged parts of the root of hair follicle. In hair follicle, blue area represented the negative for ZAP70 in young control group; brown area represented the positive for ZAP70 in other groups. Objective magnification in 200.

To confirm the localization of ZAP70 in skin, we performed the ZAP70 antibody to label its location in the mice skin by immunohistochemistry. Immunohistochemistry result shows that ZAP70 was ubiquitous localized in corium, especially in the root of hair follicle (Figure 2).

**Figure 3:**
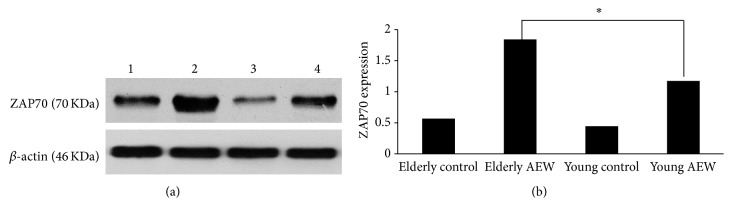
Western blotting analysis showed the expression of ZAP70 protein in skin. (a) showed ZAP70 protein levels. Lane 1 represented elderly control group; lane 2 represented elderly AEW group; lane 3 represented young control group; lane 4 represented young AEW group. *β*-actin was used as a housekeeping protein. (b) Histogram represented the expression of ZAP70 protein levels in the skin of young group and elderly group.  ^*∗*^Represented statistic difference of protein level between elderly AEW group and young AEW group (*P* < 0.05).

The protein levels of ZAP70 expression were tested by western blot. Western blot data analysis shows that ZAP70 protein immunoreactive band was observed at about 70 KD from the shaved skin tissue, and *β*-actin (42 KD) was used as an internal control. The ratio of mean optical density (MOD) of total ZAP70 expression in model group was obviously higher than control group. The protein expression of ZAP70 in elderly AEW group was statistically significantly increased compared to young AEW group (1.84 ± 0.091 versus 17 ± 0.06, *P* < 0.05, Figure 3).

**Figure 4:**
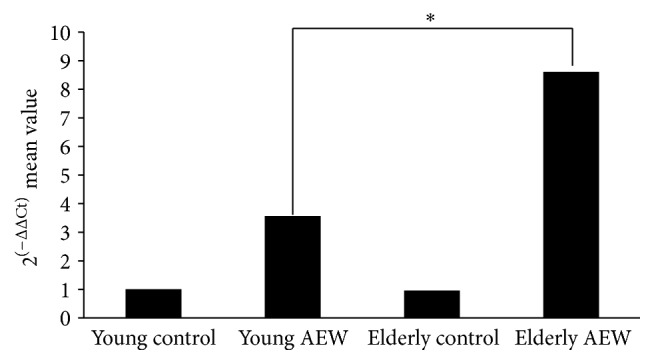
Real-time quantitative PCR displayed the mRNA levels of ZAP70 expression. Histogram revealed the gene levels of ZAP70 in the skin of young group and elderly group.  ^*∗*^Represented statistic difference of mRNA level between elderly AEW group and young AEW group (*P* < 0.05).

Moreover, we used real-time PCR to test the mRNA levels of ZAP70. The results of real-time PCR showed a similar trend with western bolt for ZAP70. The mRNA level of ZAP70 in elderly AEW group was increased compared to young AEW group (8.61 ± 0.20 versus 3.58 ± 0.18, *P* < 0.05, Figure 4).

### 3.3. IL-2 and NGF Were Increased in the Skin in Elderly Mice with Dry Skin Pruritus

**Figure 5:**
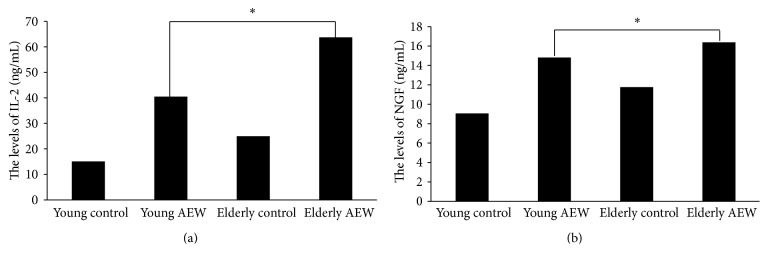
IL-2 and NGF ELISA Kits were used to test the cytokines secretion. (a) represented the IL-2 secretion in skin of young and elderly group. (b) represented the NGF secretion in the skin of young and elderly group.  ^*∗*^Represented statistic difference of cytokines levels between elderly AEW group and young AEW group (*P* < 0.05).

Furthermore, we used IL-2 and NGF high sensitivity mice ELISA Kit to test the cytokines secretion in skin between young and elderly mice. The ELISA data analysis shows that the levels of IL-2 in skin in elderly AEW group have an obvious increase compared to young AEW group (63.73 ± 1.92 versus 40.47 ± 1.57, *P* < 0.05). Also, NGF secretion in elderly AEW group was increased in skin compared to young AEW group (16.38 ± 0.28 versus 14.82 ± 0.28, *P* < 0.05) (Figure 5).

## 4. Discussion

Our results reveal that elderly mice have a more serious pruritus than young mice. Subsequently, we find that ZAP70 expression in elderly AEW group is statistically increased compared to young AEW group. The higher expression of ZAP70 in skin may explain the mechanism of dry skin pruritus in elderly. Also, IL-2 and NGF secretion are increased in elderly group compared to young group.

The AEW process induced continuous changes in the function and lesions of the skin, which indeed correlate with increased activation-regulated chemokine (TARC) level [16]. The increased level of TARC contributes to the recruitment of lymphocytes, monocytes, and polymorphonuclear cells to the skin [17]. According to recent research, TARC is involved in T-cell migration which is associated with dry skin pruritus [18]. With the increased lymphocytes of recruitment, the expression of ZAP70 is increased in skin. Then increased ZAP70 activates T-cell signals and promotes cytokines secretion.

Previous studies demonstrate that ZAP-70 reconstitute induction of IL-2 promoter activity, which regulate for IL-2 secretion and produced high level of IL-2 [19, 20]. IL-2 is used in the treatment of metastatic cancer but induced severe pruritus [21–23]. Moreover, IL-2 promotes T-cell differentiation into Th2 cells and suppressed Th1 cells, which play an important pathogenetic role in pruritic dry skin diseases [24]. Based on these discussions, the increased ZAP70 cause IL-2 increased and severe pruritus. As for the increased level of NGF, recent study finds the correlations between levels of NGF and TARC in cutaneous T-cell lymphoma (CTCL) patients, who experience severe pruritus. NGF associate with increased dermal nerve fibers and induce pruritus which may be via TARC. The enhanced NGF expression by keratinocytes and increased dermal nerve fibers are detected in lesion skin [25]. Also, hyperinnervation is considered as one cause of itch sensitization.

Age has a significant impact on dry skin pruritus. Firstly, aging is related to physiological metabolic changes, affects all persons, and is inevitable [26]. It is characterized by the thinning of the upper cutaneous layers, a decrease in the amount of lipids, diminished blood flow, and fragmentation of collagen [26]. Decreased sebaceous and sweat gland activity combined with impaired skin permeability leads to dry skin (xerosis), which is extremely common in elderly subjects. Secondly, previous studies demonstrate that after AEW process treatment the barrier recovered more slowly in aged skin [27]. Based on the previous studies, elderly mice have more susceptibility to irritant than young mice and the continuous changes in lesions may be more serious in aged skin. Thus, we suppose that elderly skin conditions induce a more serious pruritus and a high level of TARC secretion.

In conclusion, our study demonstrates that increased ZAP70 participates in the development of dry skin in elder pruritus via secretion of IL-2 and NGF.
